# CD3D, GZMK, and KLRB1 Are Potential Markers for Early Diagnosis of Rheumatoid Arthritis, Especially in Anti-Citrullinated Protein Antibody-Negative Patients

**DOI:** 10.3389/fphar.2021.726529

**Published:** 2021-09-16

**Authors:** Junqin Lu, Yihui Bi, Yapeng Zhu, Shi Huipeng, Wenxiu Duan, Jian Zhou

**Affiliations:** ^1^ Department of Orthopedics, The First Affiliated Hospital of Anhui Medical University, Hefei, China; ^2^ Department of Orthopedics, The Second Affiliated Hospital of Anhui Medical University, Hefei, China; ^3^ Department of Interventional Radiology, The First Affiliated Hospital of USTC, Division of Life Sciences and Medicine, University of Science and Technology of China, Hefei, China; ^4^ Department of Orthopedics, Shanghai Sixth People’s Hospital, Shanghai, China

**Keywords:** rheumatoid arthritis, bioinformatics, biomarker, ACPA-negative, CD3D, GZMK, KLRB1

## Abstract

Early diagnosis and monitoring of rheumatoid arthritis (RA) progress are critical for effective treatment. In clinic, the detection of rheumatoid factor (RF) and anti-citrullinated protein antibodies (ACPA) are usually combined to diagnose early RA. However, the poor specificity of RF and high heterogeneity of ACPA make the early diagnosis of RA still challenging. Bioinformatics analysis based on high-throughput omics is an emerging method to identify novel and effective biomarkers, which has been widely used in many diseases. Herein, utilizing an integrated strategy based on expression correlation analysis and weighted gene coexpression network analysis (WGCNA), we identified 76 RA-trait different expression genes (DEGs). Combined with protein-protein interaction (PPI) network construction and clustering, new hub genes associated in RA synovia, CD3D, GZMK, and KLRB1, were identified. We verified the specificity of these genes in the synovium of RA patients through three external datasets. We also observed high sensitivity and specificity of them for ACPA-negative patients. CD3D, GZMK, and KLRB1 are potentially key mediators of RA pathogenesis and markers for RA diagnosis.

## Introduction

Rheumatoid arthritis (RA) is a systemic autoimmune disease characterized by chronic inflammation in the synovium tissue of joints (McInnes and Schett, 2011; Gibofsky, 2014; Malmstrom et al., 2017; Smolen et al., 2018). A variety of immune cells and cytokines are involved in synovial inflammation, which ultimately leads to the destruction of soft tissues, cartilage, and bones around the joints (Zhang et al., 2019). Early RA clearly begins months to years when autoimmune response persists and is seronegative before it becomes a manifest polyarthritis and this is known as “preclinical RA.” The treatment of RA is usually only effective in the early stage, and many patients gradually lose their drug response as the disease progresses (Guo et al., 2018). Thus, early diagnosis is pivotal to optimal therapeutic success. Some serum biomarkers like rheumatoid factor (RF) (MacGregor et al., 2000; Onuora, 2012), anti-citrullinated protein antibody (ACPA) (Padyukov et al., 2011; Stahl et al., 2012), anti-cyclic citrullinated peptide (anti-CCP) antibody (Zendman et al., 2006), C-reactive protein (CRP) (Lee et al., 2016), and erythrocyte sedimentation rate (ESR) (Orr et al., 2018) have good performance to discriminate part of typical early RA patients, but approximately 30% of patients remain seronegative using current immunoassays. Other biomarkers, such as miRNA (Cunningham et al., 2021), calprotectin (Jarlborg et al., 2020), anti-RA33 (Syed Mohamed Suhail et al., 2019), and anti-carbamylated protein (anti-CarP) antibodies (Lamacchia et al., 2021), may be effective in the diagnosis of early RA, but these markers are not widely accepted in clinical application, and their prognostic significance remains controversial owing to the lower sensitivity and specificity of supplementary diagnosis compared with RF and ACPA. Exploring hub genes and their expression status in the inflamed synovium is a critical step in defining new targets for the diagnosis or treatment of RA, especially for the ACPA-negative RA patients whose condition could not be well managed at the developing stage due to negative index of clinical symptoms and serologic testing.

Application of transcriptomic or microarray analysis to whole synovial tissue has already identified specific genes associated with RA (Lewis et al., 2019). However, in most studies, there are many shortcomings such as small sample size, high sample heterogeneity, and only using a single technology platform and other drawbacks exist in most studies. By effectively integrating new high-throughput data, especially gene expression and proteomic-profiling data, bioinformatics analysis is expected to deliver novel clinical diagnostic insights and therapeutic options at high resolution in an unbiased fashion (Sirota and Butte, 2011; Okada et al., 2014; Stephenson et al., 2018). This strategy has already been used to discover distinct transcriptomic features of synovial fibroblasts and found that MTF1 and RUNX1 may be crucial future targets for RA therapy (Tsuchiya et al., 2020). Bioinformatics analysis also defined anti-PTX3 and anti-DUSP11 autoantibodies as newly identified biomarkers for ACPA-negative RA diagnosis (Li et al., 2021).

Herein, aiming to investigate some new and effective biomarkers for improving the management of early diagnosis of RA, especially for ACPA-negative patients, we integrated multiple databases from different GEO platforms to deeply analyze the characteristic genes of synovial tissue for RA patients. Combining differential expression analysis and weighted gene coexpression network analysis (WGCNA), we screen out 76 RA-trait different expression genes (DEGs). Followed by protein-protein interaction (PPI) network construction and hub genes selection, we identified CD3D, GZMK, and KLRB1 as three novel hub genes with RA characteristics. Independent dataset verification indicated that CD3D, GZMK, and KLRB1 can well distinguish RA patients from normal and osteoarthritis (OA) patients and has high sensitivity and specificity for ACPA-negative RA patients.

## Materials and Methods

### Microarray Data Preparation

Gene expression profiling datasets were obtained from the NCBI-GEO database (https://www.ncbi.nlm.nih.gov/gds). Datasets GSE55235 and GSE55457 originating from GEO platform 96 (GPL96) were combined with GSE77298 and GSE153015 datasets originating from GEO platform 570 (GPL570) for subsequent analysis. The four datasets contained 100 joint synovial tissue samples, including 27 normal joint synovial samples, 24 OA joint synovial samples, and 49 RA joint synovial samples. Additionally, three datasets were chosen for verification, which included GSE39340 dataset originated from GPL10558, GSE55584 dataset originated from GPL96, and GSE89408 dataset originating from GPL11154. These three datasets contained 202 joint synovial tissue samples, of which 28 are normal, 22 OA, and 152 RA synovial tissues (Table 1).

**TABLE 1:** Selected GEO datasets for bioinformatics analysis.

GEO datasets	Platform	Samples			Data category	Application
		Normal	OA	RA		
GSE55235	GPL96	10	10	10	Expression profiling by array	Analysis
GSE55457	GPL96	10	10	13	Expression profiling by array	Analysis
GSE77298	GPL570	7	0	16	Expression profiling by array	Analysis
GSE153015	GPL570	0	4	10	Expression profiling by array	Analysis
GSE39340	GPL10558	0	7	10	Expression profiling by array	Verification
GSE55584	GPL96	0	6	10	Expression profiling by array	Verification
GSE89408	GPL11154	28	22	152	Expression profiling by high-throughput sequencing	Verification

### Integration and Normalization of Microarray Data

The raw data were downloaded and preprocessed before analysis. Background correction, prosummarization, and missing values supplement for the matrix data of each GEO dataset were performed systematically by the “affy” package and the “impute” package in R/Bioconductor software (version 4.0.3). The combat function of “sva” package was used to correct the batch effects between different datasets. Principal component analysis (PCA) construction via “factoextra” package was performed to discover whether the batch effect was eliminated.

### Identification and Analysis of DEGs

Based on the comparison of gene expression values from the RA vs normal groups and the RA vs OA groups, the “limma” package in R was used to identify DEGs. The overlapped DEGs were outlined as RA-trait DEGs when adjusted *p* < 0.05 and |logFC|>1 were used as the filter criteria. Furthermore, the DEG expression levels were visualized in heatmaps and volcano plots by the “pheatmap” and “RColorBrewer” packages in R.

### Construction of WGCNA

The merged gene matrix was loaded and checked to exclude abnormal samples which might be escape from sample clustering. The merged dataset contains 100 samples and 12,412 genes for further WGCNA analysis. After selecting an appropriate threshold, adjacency and topological overlap matrix (TOM) were established to be a nearly no-scale network. Then, different modules were recognized through dynamically tree cutting with the calculation of cluster dendrogram. According to the significance of the gene coexpression network, gene significance (GS) and module membership (MM) were obtained to investigate specific modules highly related to clinical traits of RA. Finally, according to the threshold values |GS| > 0.5 and |MM| > 0.7, significant RA-correlated genes were selected as RA-trait module genes.

### Identification of RA-Trait DEGs and Gene Ontology/Kyoto Encyclopedia of Genes and Genomes (GO/KEGG) Enrichment Analyses

The overlapped genes calculated by TBtools (version 1.089) between RA-related DEGs and RA-trait module genes were featured as RA-trait DEGs. GO and KEGG pathway analyses were performed via “clusterProfiler” package in R to acquire the enriched cellular component (CC), biological process (BP), molecular function (MF) categories, and functional pathways. The significant enriched functions and pathways were filtered with adjusted *p* < 0.05 and visualized in bubble plots executed by the “ggplot2” package in R.

### Construction of PPI Network and Key Genes Analysis

The PPI network was constructed via the online tool STRING (https://string-db.org/) based on the RA-trait DEGs. Cytoscape (version 3.8.2) was applied for the better presentation and visualization of the whole interaction information. The most important cluster in the PPI network was identified by Minimal Common Oncology Data Elements (MCODE), and the key genes were further screened by cytoHubba (Cytoscape plugin), which provide 12 different algorithms to rank the importance or core degree of genes.

### Box Plot Drawing and Statistical Methods

Gene expression box plots in different data sets were drawn through “ggplot2” package. Wilcoxon test was used between two variables, and the Kruskal–Wallis test was used between multiple variables.

### Analysis of Hub Genes Expression and Drawing Receiver Operating Characteristic (ROC) Curve in External Databases

The “ggplot2” and “ggpubr” packages in R were applied for genes expression analysis via drawing boxplots according to the expression values in different validation datasets. The “pROC” package was used to draw ROC curves.

## Results

### Quality Control of Gene Expression Datasets

**FIGURE 1:**
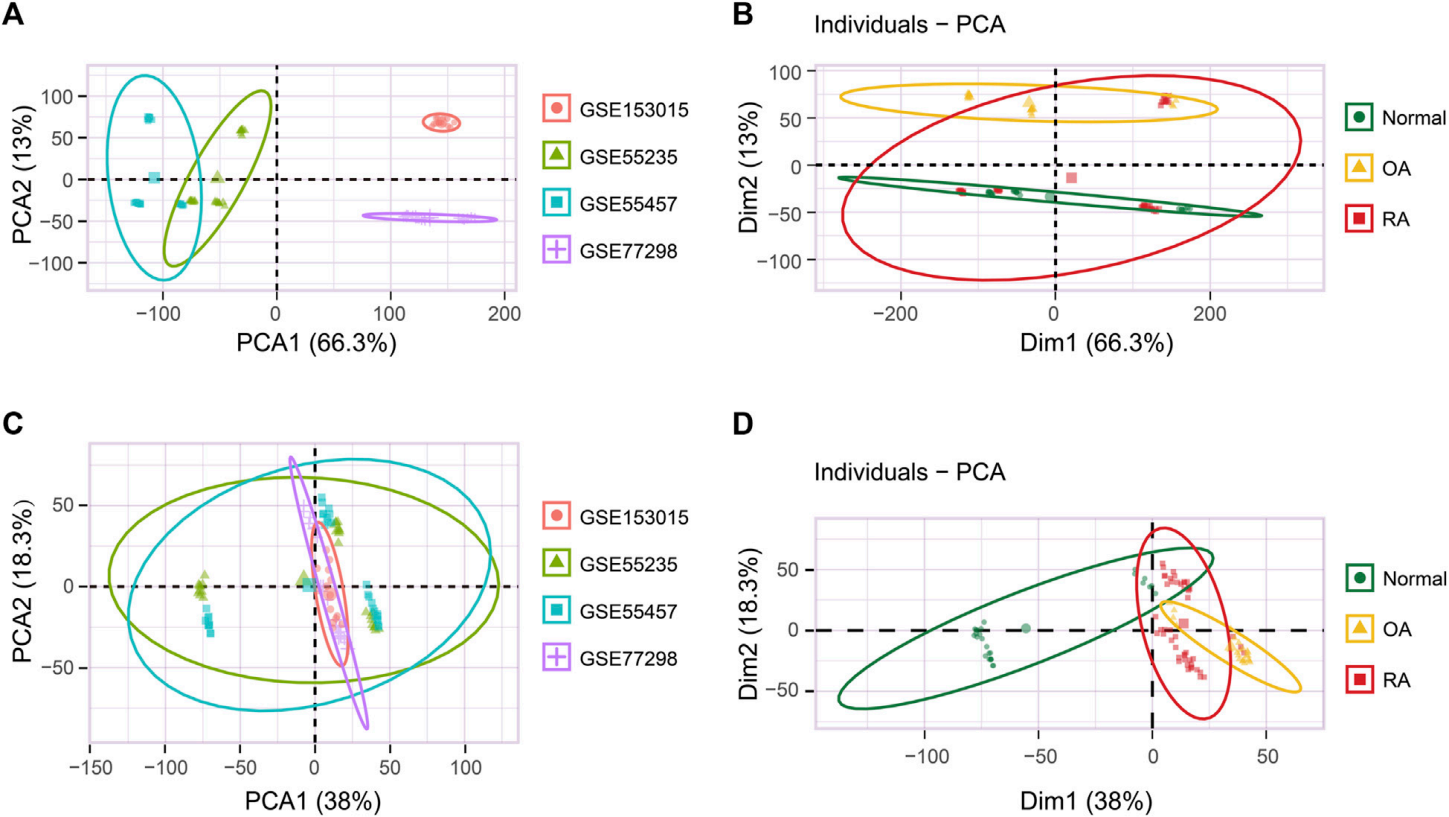
Batch-effect correction of the integrated dataset. **(A)** PCA analysis of the original integrated dataset grouped by four individual datasets. **(B)** PCA analysis of the original integrated dataset grouped by normal, OA, and RA sample types. **(C)** PCA analysis of the correcting integrated dataset grouped by four individual datasets. **(D)** PCA analysis of the correcting integrated dataset grouped by normal, OA, and RA sample types.

We determined the targeted analysis dataset integrating four independent GEO datasets originating from two GEO platforms, each employing RA, OA, and/or normal joint synovial tissue samples (Table 1). Among the GSE55235, GSE55457, GSE77298, and GSE153015 datasets for which the background correction, missing values supplement, and mean expression value calculation were performed, the batch effect was clearly observed (Figure 1A). Multivariate PCA showed that when classified according to the sample type, the integrated dataset was staggered with poor discrimination (Figure 1B). Then, the batch-effect correction was performed by the “sva” package using R, and the final data showed lower heterogeneity (Figure 1C). PCA analysis spanned 100 individuals of normal (*n* = 27), OA (*n* = 24), and RA (*n* = 49), of which three groups were clearly separated (Figure 1D). Subsequent analysis follows the principles of Supplementary Figure S1.

### Identification of DEGs in RA vs OA and RA vs Normal, Respectively

**FIGURE 2:**
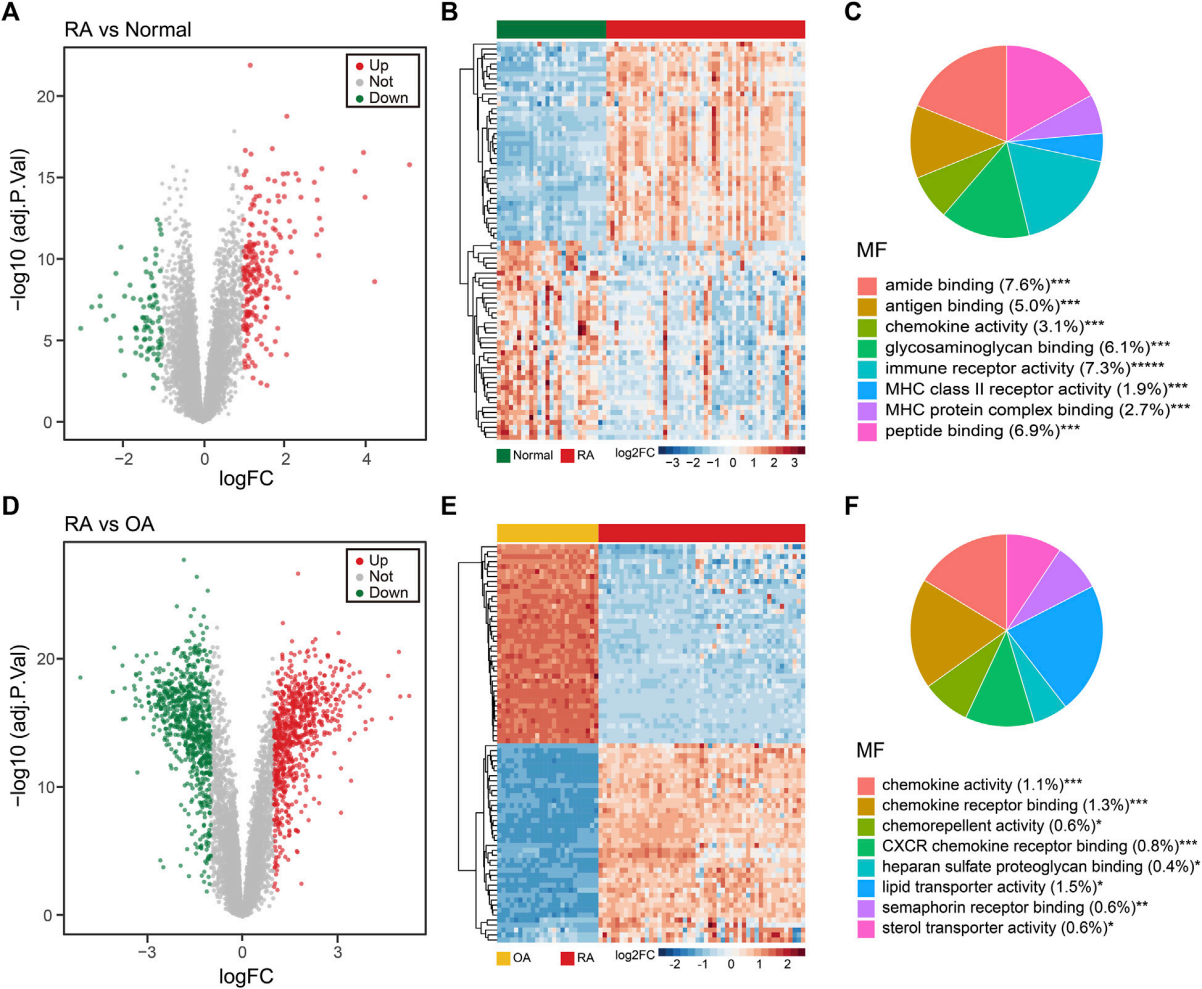
Different expression gene (DEG) analysis of the integrated dataset. **(A)** Volcano plot of the 287 DEGs between normal and RA patients. Up: upregulated; Down: downregulated; Not: no change. **(B)** Unsupervised clustering heatmap of the DEGs between normal and RA patients, top 40 were shown. **(C)** MF enrichment of GO analysis for DEGs between normal and RA patients. MF: molecular function. **(D)** Volcano plot of the 1,564 DEGs between RA and OA patients. **(E)** Unsupervised clustering heatmap of the DEGs between RA and OA patients, top 40 were shown. **(F)** MF enrichment of GO analysis for DEGs between RA and OA patients.

Next, we explored DEGs of the RA vs normal group (49 RA vs 27 normal) and the RA vs OA group (49 RA vs 24 OA). Using a *p*-value filter under 0.05 with a difference of twofold or more, we identified 287 DEGs between the RA and normal groups, of which 203 were upregulated and 84 were downregulated genes in the RA group (Figure 2A and Supplementary Table SA). In addition, we also identified 1,564 DEGs between RA and OA group, which contained 792 upregulated genes and 772 downregulated genes in the RA group (Figure 2D and Supplementary Table SB). As expected, these samples could be distinguished in an unsupervised cluster analysis based on the overall expression trend of DEGs (Figures 2B,E). GO enrichment analyses revealed that the MF of DEGs among the RA and normal groups were primarily related to amide binding, immune receptor activity, and peptide binding, whereas the main BP involved immune response-activating cell surface receptor signaling pathway, immune response-activating signal transduction, and T-cell activation. As for the CC term in the GO analysis, DEGs were mainly localized in the external side of the plasma membrane, collagen-containing extracellular matrix, and endocytic vesicle (Figure 2C and Supplementary Figures S2A,B). For the DEGs between the RA and OA groups, the MF were highly related to chemokine activity, chemokine receptor binding, and CXCR chemokine receptor binding, whereas the main BP contained leukocyte chemotaxis, myeloid leukocyte migration, and granulocyte migration. In CC analysis, DEGs were mainly localized in lipoprotein particle, plasma lipoprotein particle, and intraciliary transport particle (Figure 2F and Supplementary Figures S2C,D).

### WGCNA Identifies Critical Modules Correlating With RA

**FIGURE 3:**
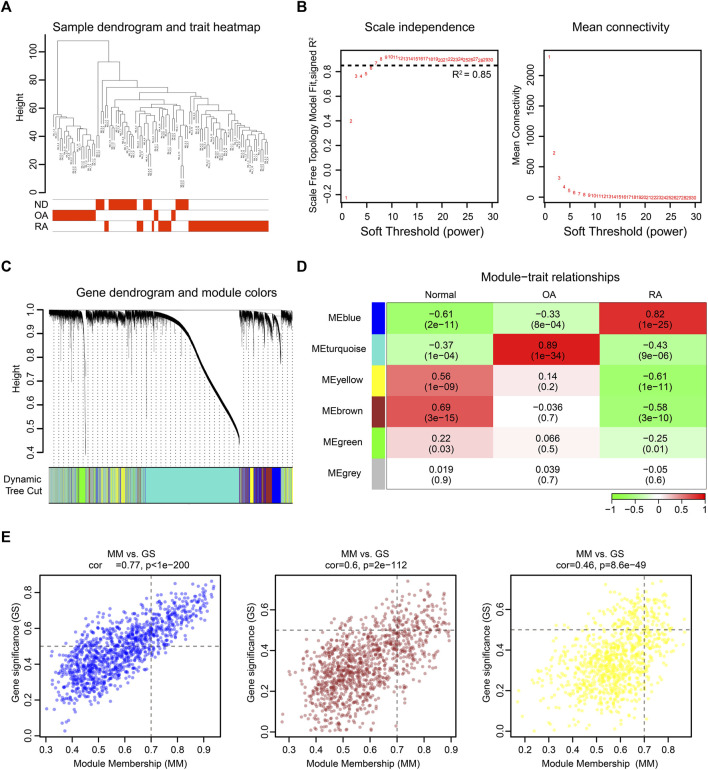
WGCNA of the integrated dataset. **(A)** Sample dendrogram and trait map. **(B)** Selection of the soft-thresholding power β. The left panel showed the scale-free fit index versus soft-thresholding power β. The right panel displayed the mean connectivity versus soft-thresholding power β. The soft-thresholding power β was selected as 7 to make the fit index curve flat (R2 > 0.85). **(C)** Gene dendrogram obtained by average linkage hierarchical clustering. The color row below the dendrogram shows the module assignment determined by the dynamic tree cut. **(D)** Model-trait relationships. Each row and column in the heatmap correspond to one module (labeled by blue, turquoise, yellow, brown, green, and grey). Besides, the green color in the heatmap represents the negative correlation, and the red color represents the positive correlation. **(E)** The scatter diagram of GS versus MM in blue, brown, and yellow modules. The gene closer to the upper right corner is more related to RA.

To further investigate the changed genes in the RA group, we performed an unbiased gene expression analysis to identify coexpressed genes and modules in our dataset. WGCNA is a systems biology method used to decipher coexpression patterns among genes across different samples (Langfelder and Horvath, 2008). In our WGCNA analysis, the integrated dataset was firstly clustered to screen out whether there were any outliers, and the sample clustering heatmap revealed that no sample was excluded (Figure 3A). The soft-thresholding power β was selected as 7, which could make the scale-free network evaluation coefficient R2 equal to 0.85 (Figure 3B). Then, we identified 5 gene coexpression modules except the grey module which incorporated uncategorized modular genes through TOM matrix hierarchical clustering and dynamic tree cut (Figures 3C,D). Moreover, the blue module (R2 = 0.82; P = 1e-25), the yellow module (R2 = −0.61; P = 1e-11), and the brown module (R2 = −0.58; P = 3e-10) were highly associated with RA. In addition, the values of GS and MM in three modules were calculated and presented with a scatter diagram (Figure 3E). Finally, by setting the threshold |GS| > 0.5 and |MM| > 0.7, we screened out 397 RA-trait module genes for further study (Supplementary Table SC).

### DEGs Correlating With RA-Trait Module Genes for Hub Gene Selection

**FIGURE 4:**
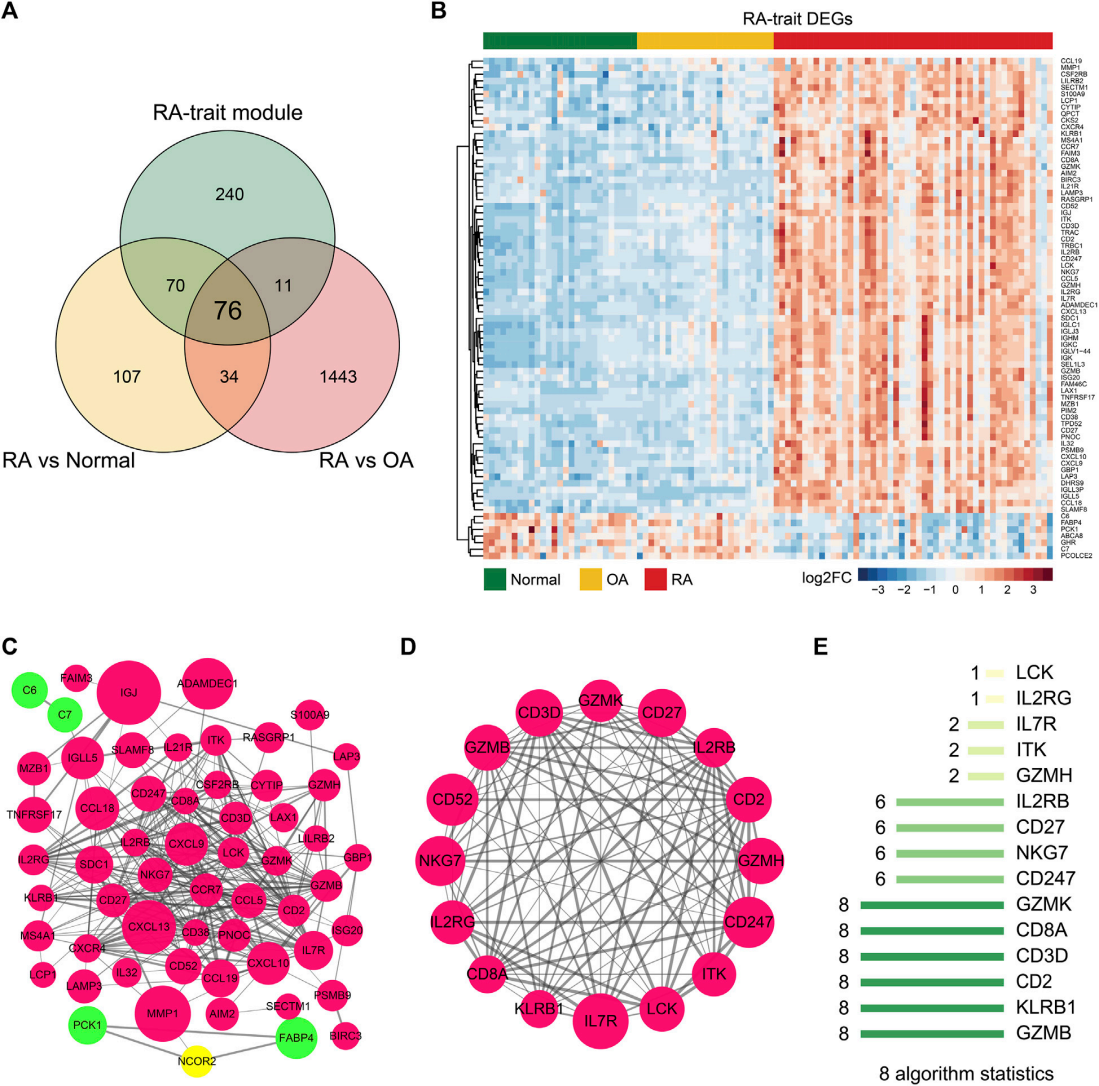
Hub genes of RA-trait DEGs selection. **(A)** Venn diagram showing gene overlap between DEGs and RA-trait module genes. 76 RA-trait DEGs were selected. **(B)** Heatmap showing unsupervised clustering analysis of RA-trait DEGs. **(C)** PPI network analysis of RA-trait DEGs. Red color represents the upregulated genes, and green color represents the downregulated genes. The yellow color represents the additional genes introduced by Cytoscape analysis. The size of the bubble is proportional to the change of expression. **(D)**. The most significant cluster containing 16 genes. **(E)** The hub genes were sorted out by 8 algorithms.

To eliminate those invalid genes which have no expression change among the RA-trait module genes, we overlapped the DEGs and RA-trait module genes by TBtools (Chen et al., 2020) (Figure 4A). 76 target genes were obtained and displayed by an unsupervised clustering analysis (Figure 4B). Go and KEGG analyses indicated that these RA-trait genes were closely related to T lymphocyte activation and regulation (Supplementary Figures S3A,B and Supplementary Tables SD, SE). The PPI network of these genes was acquired by STRING (Szklarczyk et al., 2021) and further visualized and analyzed through Cytoscape (Agg et al., 2019). As shown in Figure 4C, the PPI network in Cytoscape included 58 nodes and 287 edges (*p*-value < 1.0e-16). Then, three significant clusters were predicted through plugin MCODE (degree cutoff = 2, node score cutoff = 0.2, K-core = 2, and max. depth = 100). We observed the most significant cluster (score: 12.8) including 16 nodes and 96 edges (Figure 4D).

To sift hub genes that play a pivotal role in the related pathways, we sorted out 8 kinds of algorithms including cytoHubba, namely, BottleNeck, Closeness, Degree, EcCentricity, EPC, MCC, MNC, and Radiality to analyze the most significant cluster. The top 10 genes of each algorithm were shown in Supplementary Table SF. We observed enrichment of 6 genes including GZMB, KLRB1, CD2, CD3D, CD8A, and GZMK in all 8 algorithms, where CD2 (Raychaudhuri et al., 2009), CD8A (Carvalheiro et al., 2015; Souto-Carneiro et al., 2020), and GZMB (Bao et al., 2018) had already been reported as important genes involved in the progression of RA (Figure 4E). Finally, the three novel hub genes, CD3D, GZMK, and KLRB1 uniquely exhibited RA associations in our dimensionality reduction analysis.

### External Dataset Verification Uncovers Specifically High Expression of CD3D, GZMK, and KLRB1 in the Synovium of RA Patients

**FIGURE 5:**
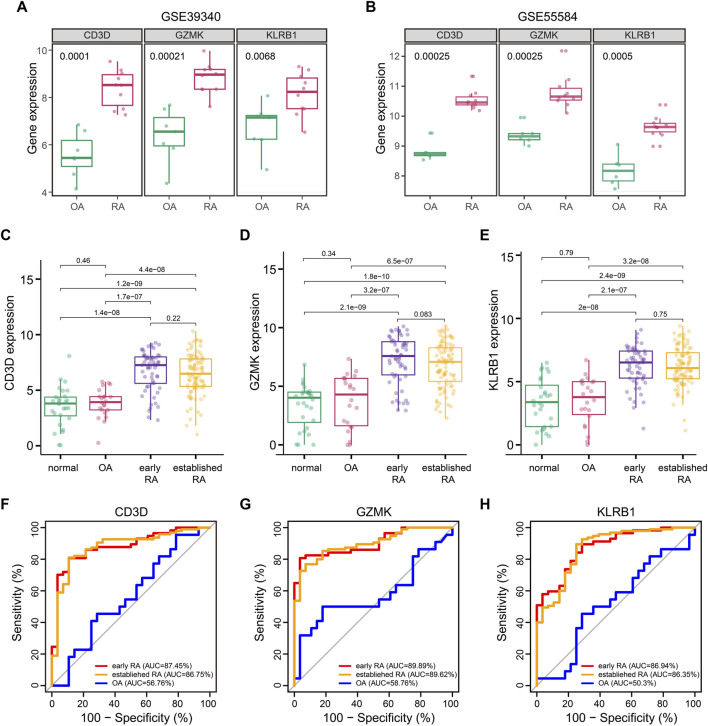
Association of CD3D, GZMK, and KLRB1 with RA. **(A)** CD3D, GZMK, and KLRB1 distribution for patients with OA and RA in GSE39340 dataset. **(B)** CD3D, GZMK, and KLRB1 distribution for patients with OA and RA in GSE55584 dataset. **(C–E)** CD3D, GZMK, and KLRB1 distribution for normal, OA, early RA, and established RA in the GSE89408 dataset. **(F–H)** Prediction of CD3D, GZMK, and KLRB1 for patients with early RA, established RA, and OA in the GSE89408 dataset.

We queried three additional datasets to validate the RA associations of CD3D, GZMK, and KLRB1. Datasets GSE39340 (GPL10558) contained 10 RA and 7 OA synovial tissue samples and another dataset GSE55584 (GPL96) contained 10 RA and 6 OA synovial tissue samples. These two gene profiling datasets both demonstrated that the three hub genes showed obvious statistical significance between RA and OA samples (Figures 5A,B). The third GSE89408 (GPL11154) dataset was a high-throughput expression profiling which contained 28 normal, 22 OA, 57 early RA, and 95 established RA synovial tissue samples. To our surprise, CD3D, GZMK, and KLRB1 not only were highly expressed in the synovial tissue of RA patients but also showed the same trend in early RA patients (Figures 5C–E).

Based on verification results, we hypothesized that CD3D, GZMK, and KLRB1 might be involved in the early evolution of RA and could become potential diagnostic markers. ROC curves were performed to assess the utility of CD3D, GZMK, and KLRB1 to differentiate between OA, early RA, and established RA. As shown in Figures 5F–H, the significant clinical correlation was only presented in early RA and established RA patients (*p* < 0.001), which indicated that these three hub genes specifically correlated with RA. The area under the curve (AUC) of the ROC curve is an indicator combining sensitivity and specificity, which could demonstrate the intrinsic effectiveness of diagnostic tests. The AUC of CD3D, GZMK, and KLRB1 levels for early RA was 0.869, 0.875, and 0.899, respectively. These three hub genes showed significantly greater predictive power for RA patients than OA patients but failed to distinguish between early RA and established RA patients.

### Performance of CD3D, GZMK, and KLRB1 in ACPA-Negative RA Patients

**FIGURE 6:**
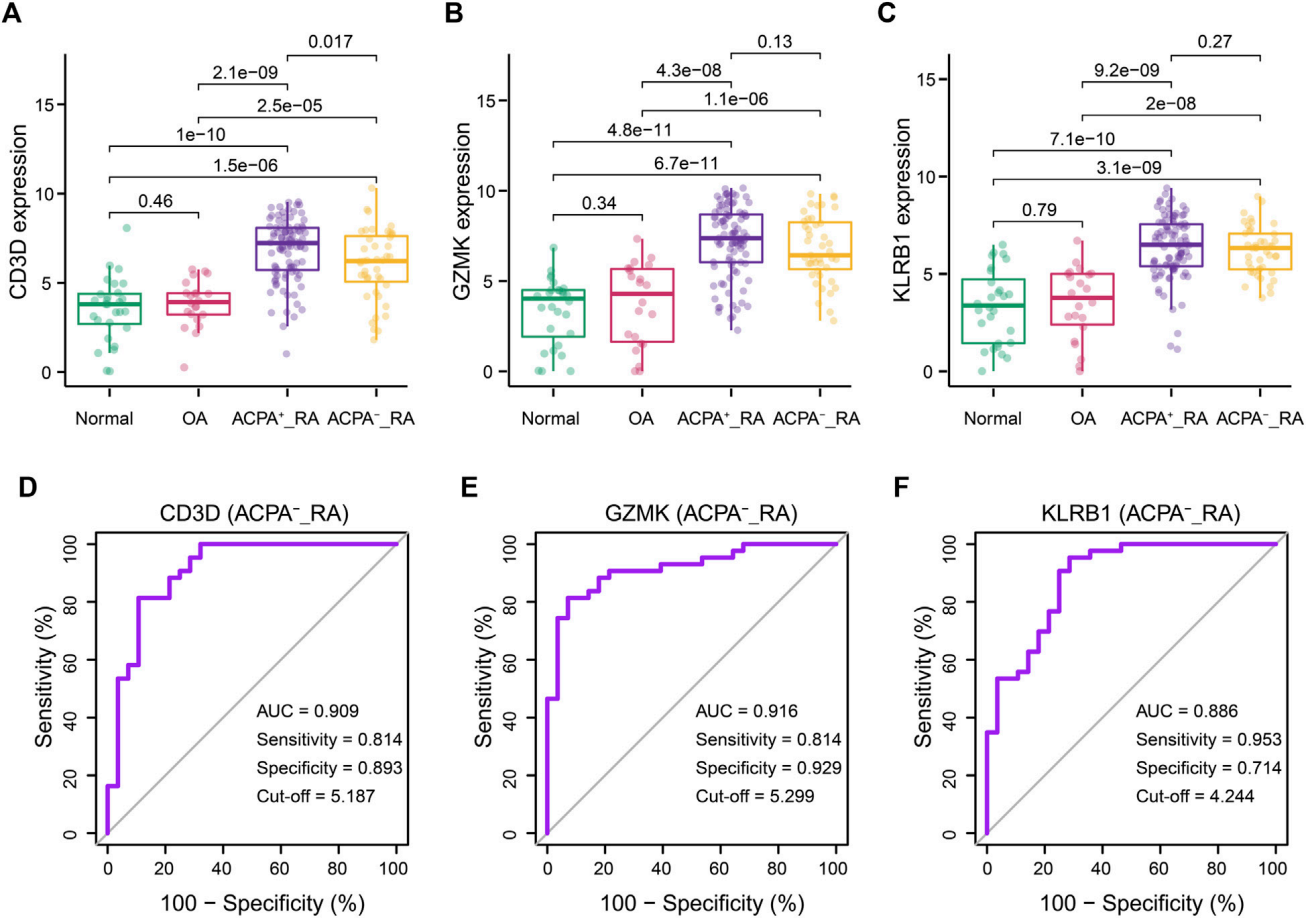
Correlations between CD3D, GZMK, KLRB1, and ACPA-negative RA diagnosis. **(A–C)** CD3D, GZMK, and KLRB1 distribution for RA patients with ACPA-positive and ACPA-negative. Normal and OA groups served as control. **(D–F)** Prediction of diagnosis value for ACPA-negative RA patients according to CD3D, GZMK,and KLRB1 expression. ACPA-_RA: ACPA-negative RA.

Clinically, ACPA-negative RA poses a great challenge for early diagnosis (Ohmura et al., 2010; Daha and Toes, 2011; Li et al., 2021). Thus, we reintegrated across the GSE89408 dataset to evaluate the performance of the CD3D, GZMK, and KLRB1 in the diagnosis of ACPA-negative RA. The RA samples in GSE89408 dataset were divided into ACPA-negative (n = 96) and ACPA-positive RA (*n* = 43) subgroups for further verification. Surprisingly, similar to the ACPA-positive RA group, the expression levels of CD3D, GZMK, and KLRB1 in the ACPA-negative RA group were significantly higher than those in the normal and OA groups (Figures 6A–C). The AUC of CD3D, GZMK, and KLRB1 expression levels was 0.909, 0.916, and 0.886, respectively (Figures 6D–F).

## Discussion

Bioinformatics analysis methods are widely used in many studies for various diseases (Kanehisa and Bork, 2003). In this study, we performed WGCNA and differential expression analysis to obtain RA-trait DEGs. GO and KEGG analyses revealed the pathological mechanism of these genes involved in the pathogenesis of RA. Six hub genes, named CD2, CD3D, CD8A, GZMB, GZMK, and KLRB1, were acquired through 8 algorithms. ROC curve revealed the diagnostic and prognostic significance of those hub genes in this study. By reviewing previous studies, GZMB, CD8A, and CD2 had been proven to have potential diagnosis and application value in RA. The other three hub genes, CD3D, GZMK, and KLRB1, were currently found to be lack of attention in RA. They were further verified in independent GEO datasets and the statistically significant difference was only presented in RA samples when compared with normal and OA samples. Meanwhile, clinical correlation analysis and ROC curve drawing were carried out and it was found that these three hub genes were not only meaningful for the early diagnosis of RA but also have guiding significance for the diagnosis of ACPA-negative patients.

Previous studies have shown that CD3D encodes the δ subunit of transmembrane CD3 antigen complex and forms the T-cell receptor/CD3 complex (TCR/CD3 complex) with the other four CD3 subunits for T-cell development and signal transduction (Gil et al., 2011). The deficiency of CD3D could cause damage to immunity (Dadi et al., 2003). Additionally, studies have reported that CD3D is a molecular diagnostic marker for immunodeficiency in early infancy (Kwan et al., 2014). Moreover, CD3D has been confirmed to actually participate in the abnormal activation of T lymphocyte immune-related pathways based on epigenetic and genomic analysis (Limbach et al., 2016; Deng et al., 2019). Coincidentally, these pathways were observed to be enriched in the present study (Supplementary Figure S4A).

GZMK is a member of the serine-proteases family, which is mainly expressed by T lymphocytes (Supplementary Figure S4B). In addition to the cytotoxicity of this family, it also has the effect of promoting proinflammatory cytokines release (Joeckel et al., 2011). Moreover, in human infectious diseases, GZMK has been found to activate protease-activated receptor-1 (PAR-1) in endothelial and fibroblast cells and induce the production of inflammatory cytokines, such as TNF-α, IL1, IL-6, and MCP-1 (Sharma et al., 2016; Herich et al., 2019). All these cytokines could cause inflammation cascades, leading to more inflammatory cells infiltration. Besides, its protease effect can promote the degradation of the extracellular matrix, resulting in inflammatory cell infiltration and tissue destruction (Turner et al., 2019). These studies suggest that GZMK may trigger the continuous inflammation amplification of RA.

The protein NKRP1A encoded by KLRB1 was a member of the NKRP1 family, and it was mainly expressed on T lymphocytes and natural killer (NK) cells (Supplementary Figure S4C). NKRP1A plays an inhibitory role in NK cell cytotoxicity (Kurioka et al., 2018). In addition, activation of NKRP1A on T cells had been found to be associated with IL17, IFN-γ, and TNF production (Billerbeck et al., 2010) and is involved in the process of inflammation and the pathogenesis of autoimmune diseases. The signal transduction mechanism of KLRB1 might be related to the activation of PI3 kinase/AKT and ERK1/2 pathways in NK cells and the PI3 kinase/AKT and STAT3 pathways in T cells (Pozo et al., 2006; Bai et al., 2014). Consistently, the activation of these signaling pathways is closely related to the pathogenesis of RA.

The study is the first to directly link the expression change of three genes with RA through unbiased and independent bioinformatics analysis, and we defined these three genes as RA-trait DEGs. We observed heterogeneity of CD3D, KZMK, and KLRB1 detectable in normal, OA, and RA synovial tissues. The gene expression of these RA-trait DEGs can distinguish early RA from OA patients. Lack of effective biomarkers in ACPA-negative RA patients impedes early diagnosis and treatment. Statistically, about one-third of RA patients are ACPA-negative, and the clinical characteristics of ACPA-negative RA are different from those of ACPA-positive RA. Our ROC curves analysis suggested that the identified RA-trait DEGs might be potential markers for ACPA-negative RA diagnosis. However, this study has several limitations. First, the specificity of this gene expression in other inflammatory or immune diseases, such as psoriasis, SLE, and multiple sclerosis, has not been investigated. Second, the sample size of our study was still limited, and it is essential to interrogate the gene expression in synovium by further experiment. Third, it is necessary to explore the possibility of checking these three genes or corresponding proteins through blood or joint fluid. In conclusion, our study could provide a theoretical basis for better management of early diagnosis of RA.

## Data Availability

The datasets presented in this study can be found in online repositories. The names of the repository/repositories and accession number(s) can be found in the article/Supplementary Material.
