# Supplementary material for: CD3D, GZMK, and KLRB1 Are Potential Markers for Early Diagnosis of Rheumatoid Arthritis, Especially in Anti-Citrullinated Protein Antibody-Negative Patients
Source: Front Pharmacol. 2021 Sep 16;12:726529. doi: 10.3389/fphar.2021.726529 (PMC8483717; doi:10.3389/fphar.2021.726529)
Supplement: Supplementary file 1 [file DataSheet1.zip › Supplementary_Material.docx]

Supplementary Material

## Supplementary Figures


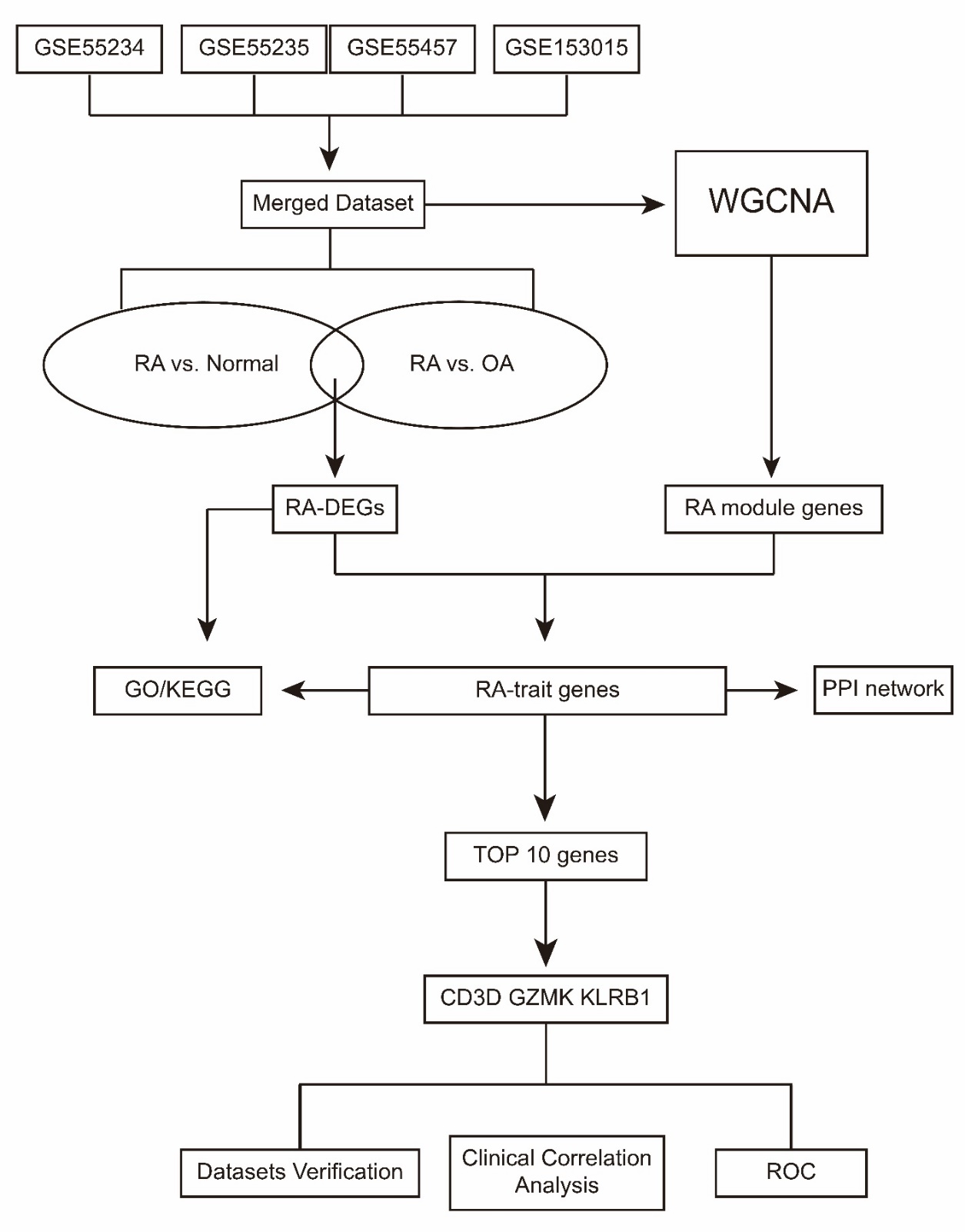


**Supplementary Figure 1.** Principles of this study.


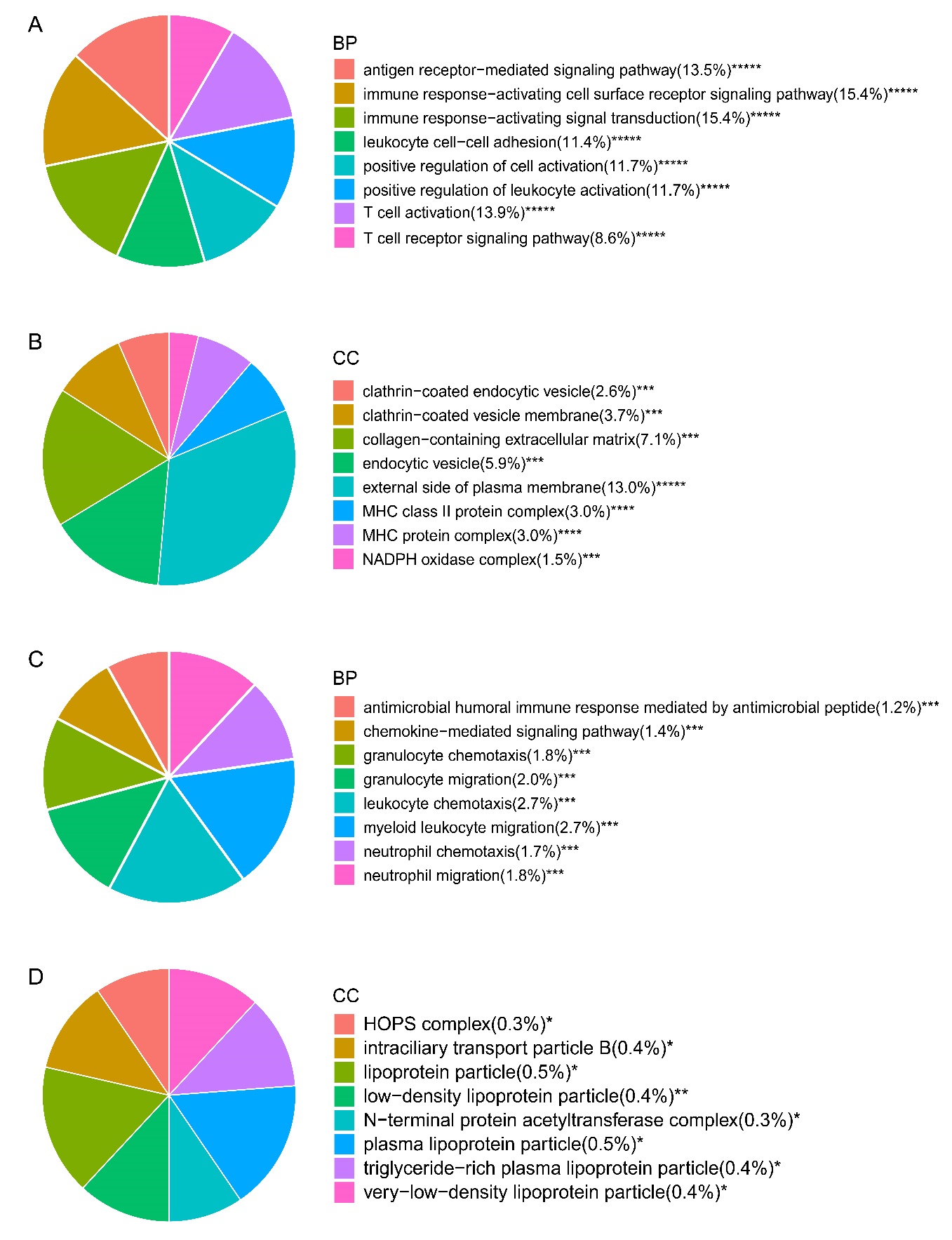


**Supplementary Figure 2.** GO analysis of DEGs. (A) Biological processes (BP) of DEGs between RA and normal groups. (B) Cellular components (CC) of DEGs between RA and normal groups. (C) BP of DEGs between RA and OA groups. (D) CC of DEGs between RA and OA groups.


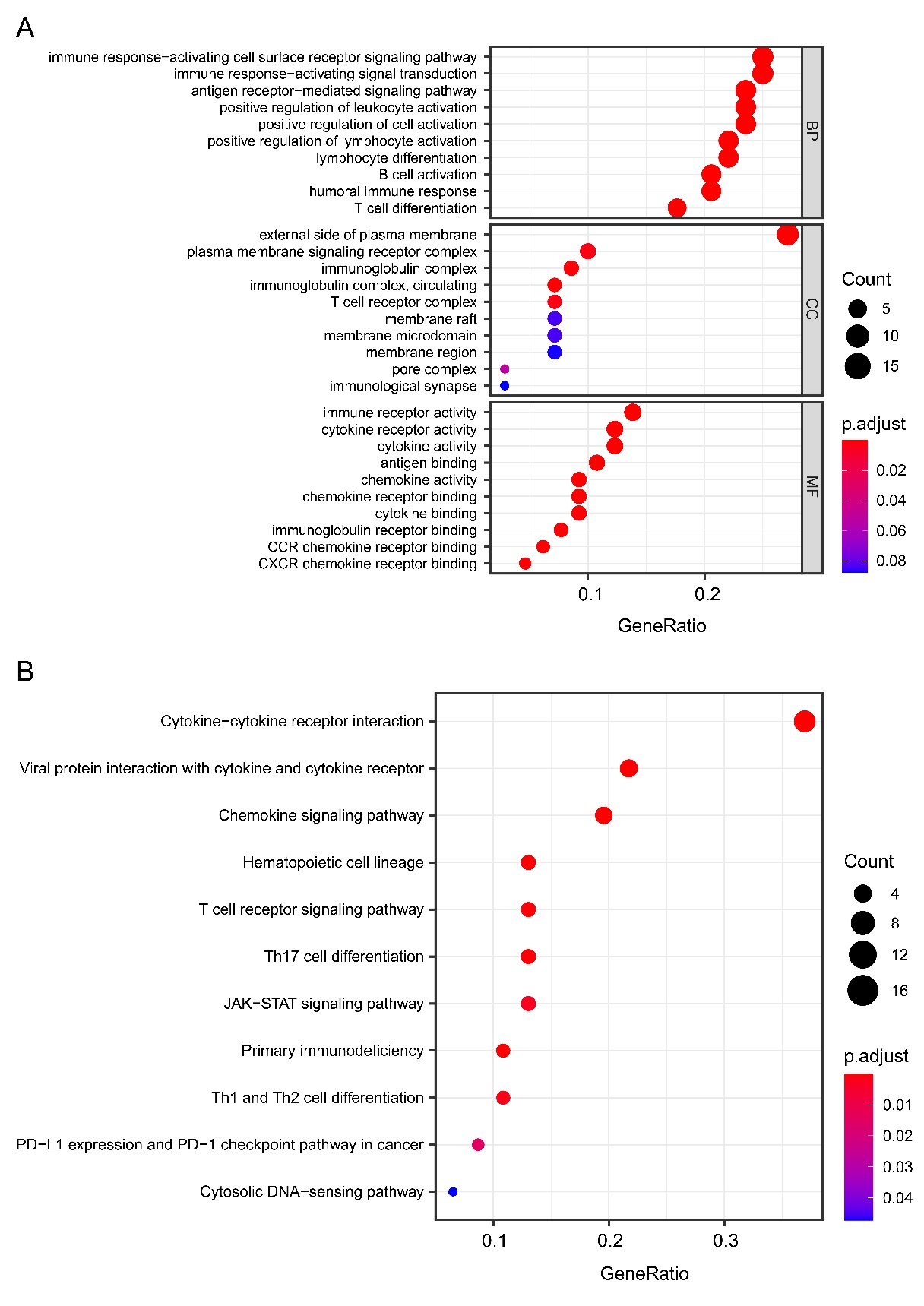


**Supplementary Figure 3.** GO and KEGG analysis of RA-trait DEGs. (A) GO analysis of RA-trait DEGs. (B) KEGG analysis of RA-trait DEGs.


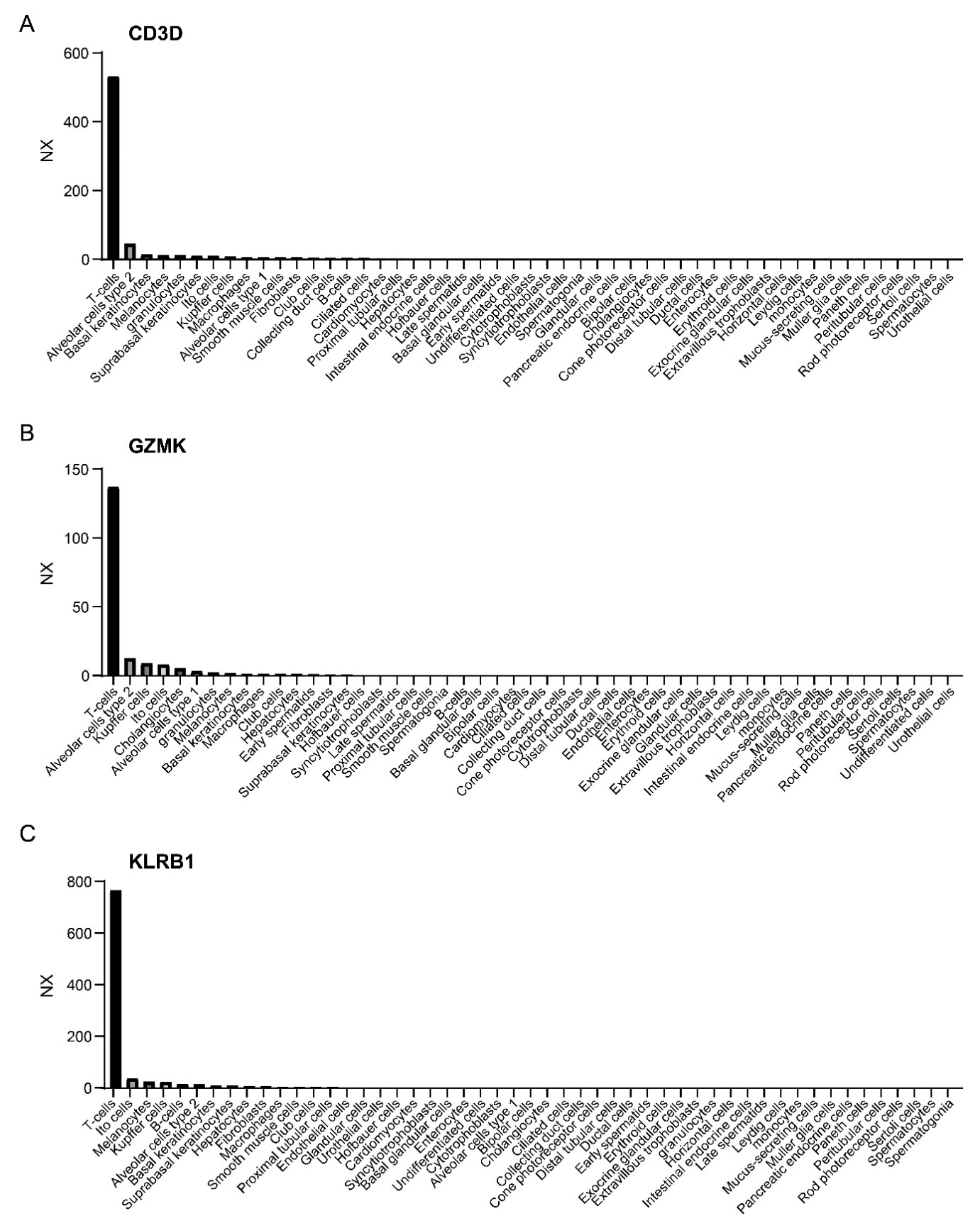


**Supplementary Figure 4.** RNA expression of (A) CD3D, (B) GZMK, and (C) KLRB1 in single cell types. The analysis was based on The Human Protein Atlas database.
